# Echocardiography-based machine learning algorithm for distinguishing ischemic cardiomyopathy from dilated cardiomyopathy

**DOI:** 10.1186/s12872-023-03520-4

**Published:** 2023-09-26

**Authors:** Mei Zhou, Yongjian Deng, Yi Liu, Xiaolin Su, Xiaocong Zeng

**Affiliations:** 1 Department of Cardiology, The First Affiliated Hospital of Guangxi Medical University, 6 Shuangyong Road, Nanning, 530021 Guangxi China; 2 Department of Cardiology, Minzu Hospital of Guangxi Zhuang Autonomous Region, Nanning, Guangxi China; 3 Guangxi Key Laboratory Base of Precision Medicine in Cardio-cerebrovascular Diseases Control and Prevention & Guangxi Clinical Research Center for Cardio-cerebrovascular Diseases, Nanning, Guangxi China; 4 School of Basic Medical Sciences, Guangxi Medical University, Nanning, Guangxi China

**Keywords:** Machine learning, Heart failure, Ischemic cardiomyopathy, Dilated cardiomyopathy, Echocardiography

## Abstract

**Background:**

Machine learning (ML) can identify and integrate connections among data and has the potential to predict events. Heart failure is primarily caused by cardiomyopathy, and different etiologies require different treatments. The present study examined the diagnostic value of a ML algorithm that combines echocardiographic data to automatically differentiate ischemic cardiomyopathy (ICM) from dilated cardiomyopathy (DCM).

**Methods:**

We retrospectively collected the echocardiographic data of 200 DCM patients and 199 ICM patients treated in the First Affiliated Hospital of Guangxi Medical University between July 2016 and March 2022. All patients underwent invasive coronary angiography for diagnosis of ICM or DCM. The data were randomly divided into a training set and a test set via 10-fold cross-validation. Four ML algorithms (random forest, logistic regression, neural network, and XGBoost [ML algorithm under gradient boosting framework]) were used to generate a training model for the optimal subset, and the parameters were optimized. Finally, model performance was independently evaluated on the test set, and external validation was performed on 79 patients from another center.

**Results:**

Compared with the logistic regression model (area under the curve [AUC] = 0.925), neural network model (AUC = 0.893), and random forest model (AUC = 0.900), the XGBoost model had the best identification rate, with an average sensitivity of 72% and average specificity of 78%. The average accuracy was 75%, and the AUC of the optimal subset was 0.934. External validation produced an AUC of 0.804, accuracy of 78%, sensitivity of 64% and specificity of 93%.

**Conclusions:**

We demonstrate that utilizing advanced ML algorithms can help to differentiate ICM from DCM and provide appreciable precision for etiological diagnosis and individualized treatment of heart failure patients.

## Background

Cardiomyopathy is the leading cause of heart failure (HF), which carries high risks of mortality and morbidity [1]. The two major types of cardiomyopathy are ischemic cardiomyopathy (ICM), which is mainly characterized by myocardial ischemia, degeneration, necrosis, fibrosis and scar formation, and dilated cardiomyopathy (DCM), which is mainly characterized by obvious enlargement of the left ventricle (LV), thinning of the ventricular wall and decreased LV systolic function [2, 3]. These two CM types have different pathophysiological characteristics but may have a similar clinical presentation, i.e., impaired LV function with disease progression to HF. Determining whether the decreased LV function in HF patients is caused by ICM due to coronary heart disease or by DCM is of great importance to early treatment planning and improving prognosis [4, 5].

At present, in studies in China and elsewhere, coronary angiography is an important technique for the differentiation of ICM and DCM [6]. However, because it is invasive examination, acceptance in the clinic is problematic, and more importantly, this technique cannot be carried out in many primary hospitals due to limited conditions. A considerable number of patients with HF and left ventricular dysfunction show clinical improvement in left ventricular function after appropriate use of coronary revascularization [7, 8]. Therefore, finding a clinically more acceptable and feasible method for the diagnosis of HF-related etiology, such as ICM and DCM, is an important objective in this field. Traditionally, noninvasive tests to distinguish the two have included electrocardiograms, echocardiograms or exercise echocardiography, chest radiographs, cardiac computed tomography (CT), cardiac positron emission tomography (PET), cardiac magnetic resonance imaging (CMRI), radionuclide studies, and genetic studies [9–11], where simple electrocardiograms are sometimes not diagnostic. In previous studies, when used to predict cardiovascular outcomes, ECG parameters have generally shown poor predictive accuracy and to possibly even reduce the incremental validity of the model [12–14]. Echocardiography is the first diagnostic step following collection of the patient’s family history, physical examination, and electrocardiography, and is crucial for the morphological diagnosis of most cardiomyopathy cases [15, 16]. Cardiac MRI is the gold standard for high-quality diagnostic imaging, if echocardiography does not clearly identify phenotypes, but the use of more advanced imaging methods is limited due to technical difficulties, high costs, and other reasons. Therefore, to avoid the influence of relevant confounding factors, this study attempted to further improve the diagnostic effectiveness of echocardiography by utilizing the power of big data.

Machine learning (ML) is a statistical learning and modeling technique that can make predictions about invisible or new data through learning from available data [17, 18]. This study aimed to use a ML algorithm based on clinical data derived from echocardiography to develop and verify a predictive model to distinguish ICM from DCM. Our novel model further improves the accuracy of diagnosis and reduces the risk of misdiagnosis, and thus, can provide practical assistance in the differential diagnosis of ICM and DCM, especially for hospitals that cannot carry out coronary angiography.

## Methods

### Study population

**Fig. 1:**
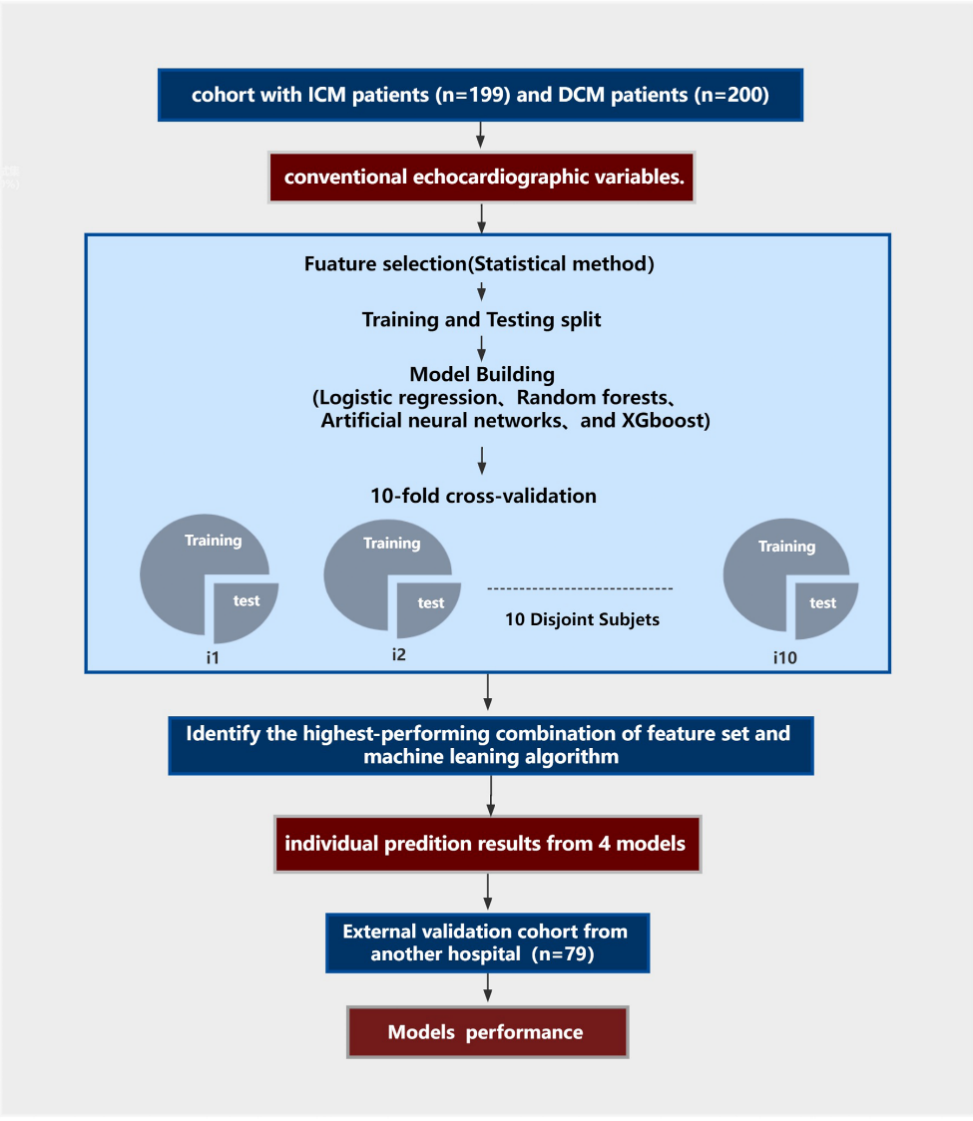
Machine learning workflow for differentiation of ICM and DCM. A machine learning model was developed to differentiate between ICM and DCM. The machine method included feature selection using a statistical method and 10-fold cross-validation, and 4 different algorithms were compared. ICM: ischemic cardiomyopathy; DCM: dilated cardiomyopathy

This study collected data for a total of 437 consecutive patients (199 with ICM and 238 with DCM) from the First Affiliated Hospital of Guangxi Medical University. The included patients were diagnosed with HF according to recently published guidelines [1]. All patients underwent invasive coronary angiography for diagnosis of ICM or DCM. DCM was defined as LV or biventricular contractile dysfunction and dilatation, rather than the presence of severe coronary artery disease and abnormal loading conditions. The diagnosis of dilated cardiomyopathy was confirmed after a systematic diagnostic procedure based on the definition of dilated cardiomyopathy published by the World Health Organization/International Society and Cardiology Federation and the latest Guidelines of China [19–21]. The diagnostic requirements were: (1) left ventricular ejection fraction (LVEF) decreased by < 45%, and left ventricular short axis shortening rate (LVFS) decreased by < 25%; (2) the left ventricular end diastolic volume or diameter of the standard map adjusted by body surface area and age was > 2 standard deviations (SDs) compared with the normal value; and (3) coronary angiography was used to evaluate coronary artery disease (CAD). However, even in the presence of CAD, the diagnosis of DCM can still be considered when the severity of HF is not proportional to the degree of CAD [22]. The inclusion criteria for ischemic cardiomyopathy were as follows: (1) history of symptomatic HF (New York Heart Association Functional grade II or higher) and a decrease in the LVEF by < 40% and (2) one or more of the following: a history of myocardial infarction or revascularization (coronary artery bypass graft or percutaneous coronary intervention), or with stenosis of 75% or more in the left main stem or left anterior descending artery, or with greater than 75% stenosis in two or more epicardial vessels [3, 20, 23]. To avoid influencing the results of the study, 38 patients with LVEF of 40–45% were excluded, and patients with missing echocardiographic and coronary angiographic data or with congenital heart disease, acute or sub-acute myocarditis, hypertrophic cardiomyopathy, primary valvular disease [24], infiltrative disease, restrictive cardiomyopathy, incomplete myocardial densification, infiltrative disease, and/or evidence of immune-mediated disease were excluded from this study. Ultimately, the study group consisted of 399 patients (199 with ICM and 200 with DCM). Figure 1 shows the patient selection and modeling process. This study was approved by the Ethics Committee of First Affiliated Hospital of Guangxi Medical University and was conducted according to the principles defined in the Declaration of Helsinki. Written informed consent was obtained from all individual participants included in this study.

### Variables

Continuous variables are presented as mean ± SD and were compared with the t-test. Categorical variables are presented as percentages and were compared with the chi-square test. Non-normal distributed data were compared with the nonparametric test. Statistical analysis was performed using the R software package (R version 3.6.2). The feature selection method and ML algorithm were realized with R software package, and a P value < 0.05 was considered statistically significant. In the final analysis, a total of 16 variables (10 continuous variables and 6 categorical variables) were included, consisting of baseline demographics (3 variables) and echocardiographic outcome parameters (13 variables), including LV end-diastolic diameter (LVEDD), left atrial diameter (LAD), LV end-systolic diameter (LVESD), LVEF, LV posterior wall thickness (LVPWT), abnormal wall motion (2 variables DWMA (diffuse ventricular wall motion abnormalities) and SWMA (segmental ventricular wall motion abnormalities)), the ratio of peak E to peak A (E/A), cardiac output (CO), LV fractional shortening (FS), stroke volume (SV), moderate to severe mitral regurgitation (MR(m-s)), and moderate to severe multiple valve regurgitation (MVR(m-s)).

### ML methodology

#### Data processing

For categorical variables, we implemented 1-hot-encoding, the process of dividing categorical values into zero and nonzero value pairs in order to convert variables into a format that can be used in the classification algorithm. In this method, the presence of missing values in variables presents a particular challenge. To handle this issue, the data with missing values for variables greater than 20% were removed, and the mean filling method was used for continuous variables with fewer missing values. Because of the fewer missing values, the filling had little impact on the overall distribution of variables. Categorical variables were predicted by combining the decision tree algorithm with other variables to obtain the predicted value to replace the missing value, which explained the statistical uncertainty related to interpolation.

**Fig. 2:**
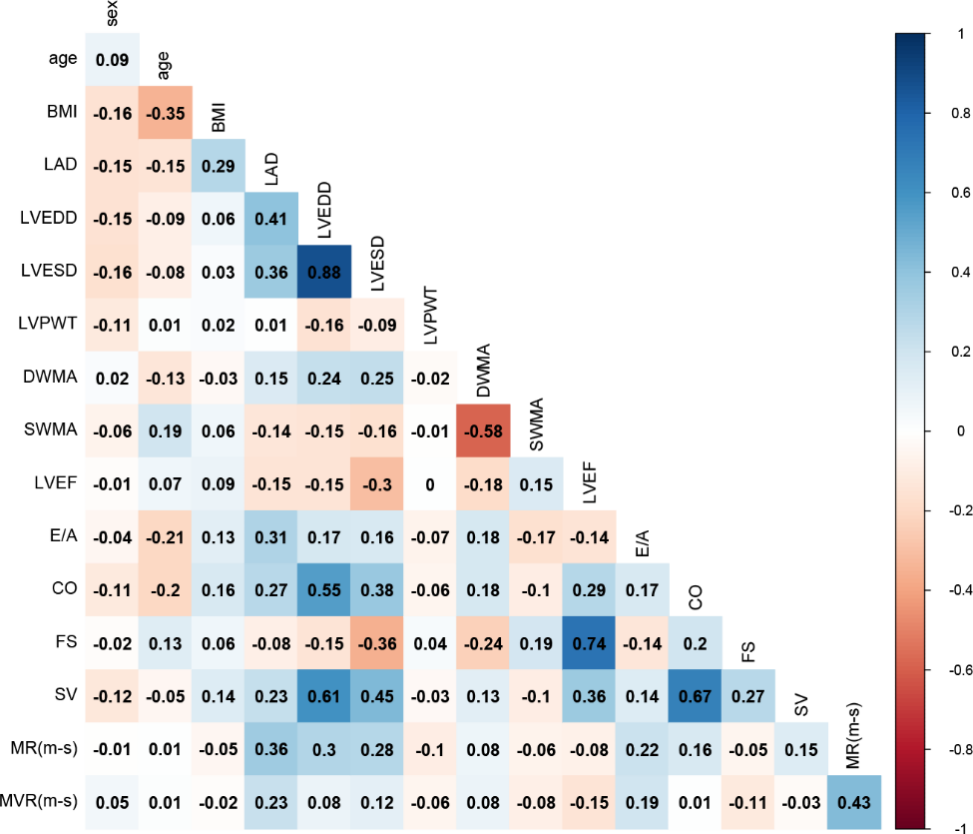
Analysis of correlations between variables in the dataset. The inclusion of highly correlated variables can lead to numerical instability, obscure the interactions between different features, affect the interpretability of machine learning models, and also lead to overfitting. Therefore, it is best to exclude one of the two relevant variables. The correlation graph shows that the dataset did not contain many relevant variables, indicating that the model is simple and stable

Prior to the model construction, value correlation of variables was performed using the R software package (R version 3.6.2) to remove variables that may cause numerical instability, which leads to over-fitting of the model and/or affect model interpretability (Fig. 2). When the linear relationship between two variables is enhanced, the correlation coefficient tends to be 1 or -1. If when one variable increases, the other variable also increases, this indicates a positive correlation between them, and the correlation coefficient will be greater than 0. In contrast, if one variable increases and another variable decreases, a negative correlation is found between them and the correlation coefficient will be less than 0. If the correlation coefficient is equal to 0, this indicates there is no linear correlation between two variables. In this case, no variables were excluded based on the correlation coefficients and clinical significance.

#### Supervised ML approach

Predictive classifiers were developed for the data of the training set using four supervised ML methods: (1) logistic regression (LR), (2) extreme gradient boosting (XGBoost), (3) random forest (RAN), and (4) artificial neural network (NNET). The above-mentioned ML-based algorithms were used in the present study, because they represented a full-spectrum analysis method from the traditional LR used with statistical analysis to traditional ML algorithms (RAN), human neuron mimetic algorithms (NNET), and integrated enhancement (XGBoost) [25]. Boosting is used with increasing frequency in ML, because it involves sequential model creation, with each iteration aiming to correct a bug in the previous model. XGBoost, which is based on a gradient enhanced decision tree. We used a grid search merging cross-validation method to adjust the hyperparameters of the XGBoost model. We used the traditional grid search for simultaneous optimization of multiple parameters and specify the range of candidate values for each parameter of the model. As for tree booster, eta can improve the robustness of the model by reducing the weight of each step. Values of 0.1 and 0.3 were selected. Min_child_weight determines the minimum leaf node sample weight and adopts the combination of 1, 6, 10, and 12. Max_depth of the tree was set to 5, 6, 10, and 16, respectively. Gamma specifies the minimum loss function drop value required for node splitting. Subsample controls the proportion of random samples for each tree. This parameter was set to be 0.5, 0.7, and 1, respectively. Colsample_bytree controls the proportion of random samples for each tree. The default value was 1. Finally, we selected 13 times, 50 times, 100 times and 200 times of iterations and carried out model cross validation through grid arrangement and combination of all parameters. Generally, 10 times of cross validation was selected and multiple parameters were selected through observation accuracy and Kappa value. Finally, relevant parameters of the model were determined as follows: nround = 13, eta = 0.1, gamma = 0, subsample = 1, max depth = 5, colsample_bytree = 1, min_child_weight = 6 to produce good performance.

In addition, to evaluate the validity of each model, we also used the K-fold cross-validation technique on a random under-sampled subset of the entire dataset. We performed 10 cross-validations by randomly dividing the entire dataset into 10 parts for 10 iterations. In each iteration, we selected 9 parts as training data and 1 part as test set. The average result was 10% of test data unused for each model. The overall performance of the predictive model on the test set was evaluated by calculating the area under the curve (AUC) from the receiver operating characteristic curve. Finally, the calibration results for each model were reported. Calibration further reflected the stability of the model.

## Results

### Patient characteristics

The demographic and echocardiographic data of all patients in the ICM (n = 199) and DCM (n = 200) groups are summarized in Table 1. The ICM patients were older (mean age 64 years, P < 0.001) and included a higher proportion of men (88.4% vs. 75.5%, P = 0.001) compared with DCM patients. ICM patients also more often had significant segmental ventricular wall motion abnormalities (57.8% vs. 13.5%, P < 0.001), with an E/A ratio less than 1, while more DCM patients had diffuse ventricular wall motion abnormalities (98% vs. 67.8%, P < 0.001). Analysis of correlation parameters such as LAD, LVEDD, and LVESD suggested that heart chamber enlargement was more significant (P < 0.001). There were also significant differences in FS and LVEF between the two groups (P < 0.001).

**Table 1:** Baseline and echocardiographic characteristics of the included patients in the ICM and DCM groups

Variable		ischemic (n = 199)	dilated (n = 200)	P
Sex (%)	man	176 (88.4)	151 (75.5)	0.001
	woman	23 (11.6)	49 (24.5)	
Age (years)		64.21 (10.58)	57.39 (12.25)	< 0.001
BMI (kg/m^2^)		23.78 (3.86)	24.36 (4.58)	0.173
LAD (mm)		43.15 (6.64)	46.24 (6.67)	< 0.001
LVEDD (mm)		65.93 (8.03)	69.52 (8.52)	< 0.001
LVESD (mm)		55.38 (7.21)	59.06 (9.54)	0.001
LVPWT (mm)		10.0[9.00,11.00]	10.00[9.00,11.00]	0.009
DWMA (%)	no	64(32.2)	4 (2.0)	< 0.001
	yes	135(67.8)	196 (98.0)	
SWMA (%)	no	84(42.2)	173 (86.5)	< 0.001
	yes	115(57.8)	27 (13.5)	
LVEF (%)		32.16 (4.96)	30.09 (5.56)	< 0.001
E/A (%)	less1	116(58.3)	69 (34.5)	< 0.001
	Single-Peaked	28(14.1)	57 (28.5)	
	more1	55(27.6)	74 (37.0)	
FS (%)		16.35 (4.05)	14.57 (3.18)	< 0.001
SV (ml/B)		71.48 (22.95)	77.59 (29.50)	0.021
MR (m-s) (%)	no	153(76.9)	127 (63.5)	0.005
	yes	46(23.1)	73 (36.5)	
MVR (m-s) (%)	no	191(96.0)	176 (88.0)	0.008
	yes	8(4.0)	24 (12.0)	
CO (L/min)		6.03 (2.00)	6.79 (2.28)	0.006

Abbreviations: BMI: body mass index; LAD: left atrial diameter; LVEDD: left ventricle end-diastolic diameter; LVESD: left ventricle end-systolic diameter; LVPWT: left ventricle posterior wall thickness; DWMA: diffuse ventricular wall motion abnormalities; SWMA: segmental ventricular wall motion abnormalities; LVEF: left ventricle ejection fraction; E/A: the ratio of peak E to peak A; FS: left ventricle fractional shortening; SV: stroke volume; MR(m-s): moderate to severe mitral regurgitation; MVR(m-s): moderate to severe multiple valve regurgitation; CO: cardiac output

### ML analysis

#### Variable selection

**Fig. 3:**
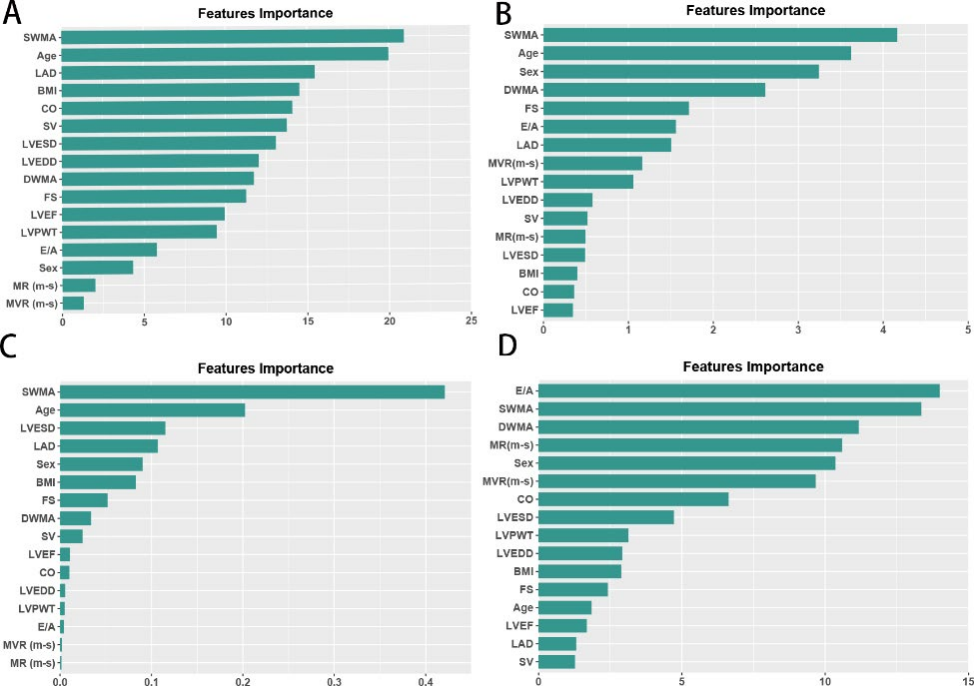
Ranking of feature importance. The importance graph for the variables was obtained after training with tuning parameters on the training data set. The most important variables in the study cohort for the differentiation of ICM and DCM are ranked. **(A)** random forest, **(B)** logistic regression, **(C)** extreme gradient boosting, and **(D)** artificial neural network

Variable importance graphs were obtained after training with tuning parameters on the training data set (90% of total cohort). Figure 3 C shows the ranking of the most significant variables in the study cohort that differentiated ICM from DCM. In the best-performing XGBoost model, segmental wall motion anomalies were the most important predictor, followed by age, LVESD and LAD. Other significant variables worth noting were sex, BMI, and FS.

#### ML model

**Fig. 4:**
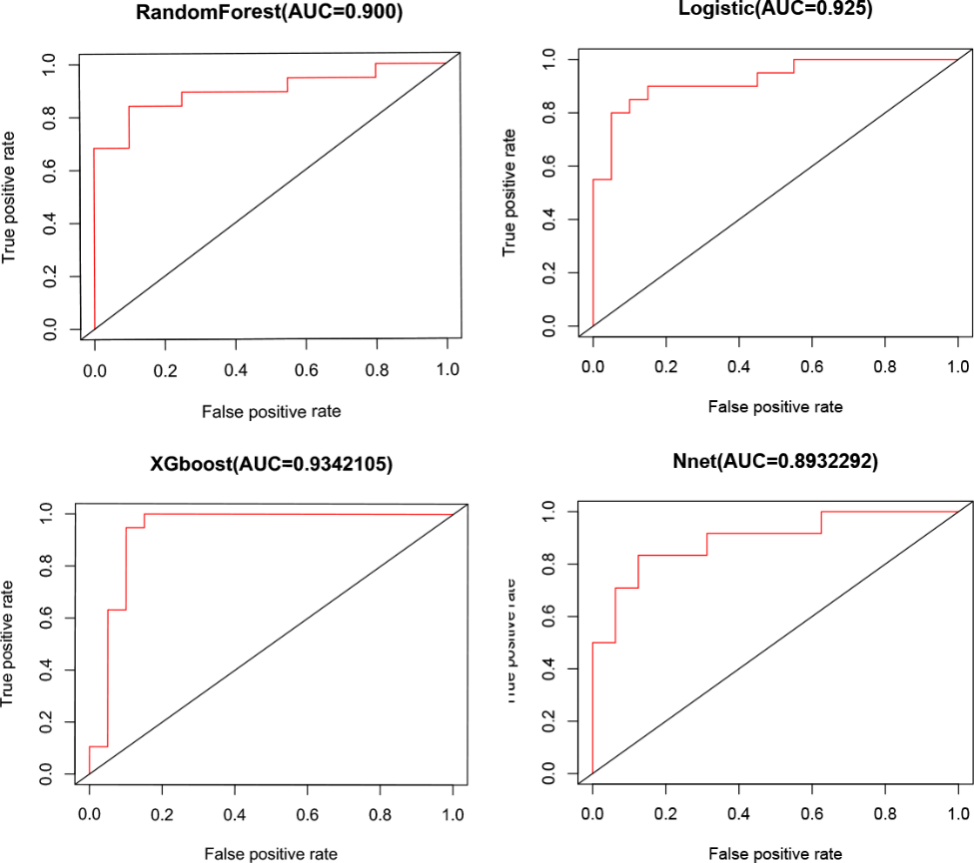
Receiver operating characteristic curves for prediction of ICM and DCM in the test sample. XGboost model presented a higher AUC for distinguishing ICM and DCM than all other models (LR, RAN and NNET). AUC: area under the curve; LR: logistic regression; NNET: neural network algorithm; RAN: random forest; XGboost: extreme gradient boosting

**Fig. 5:**
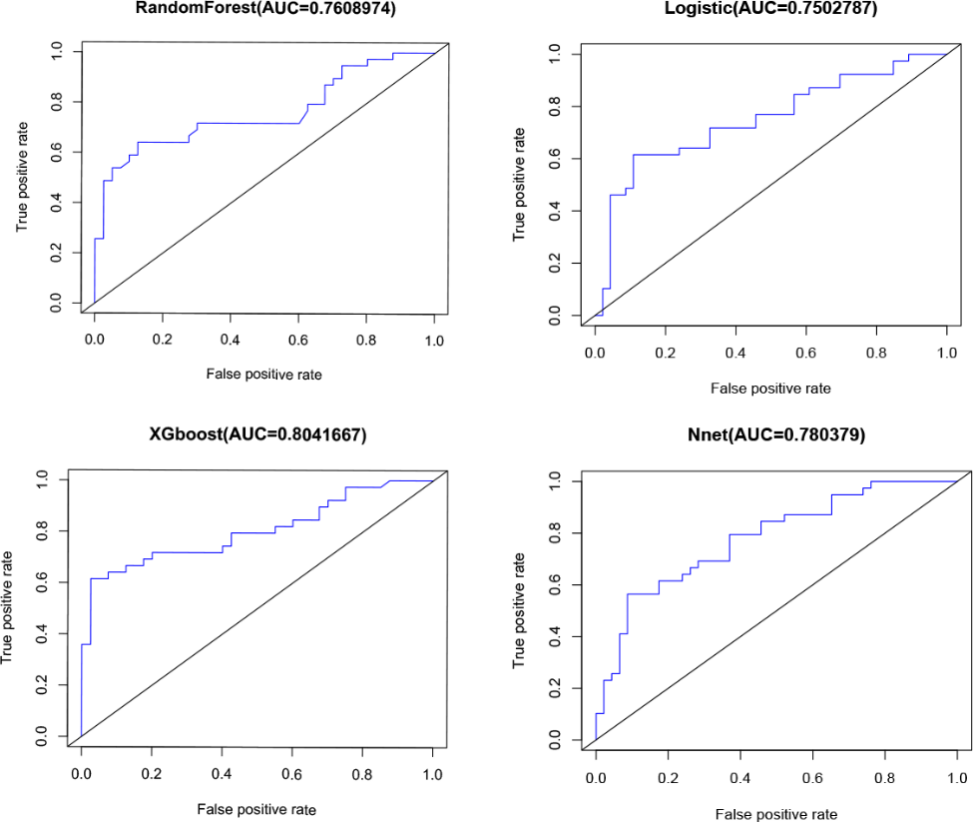
Receiver operating characteristic curves for prediction of ICM and DCM in the external validation. XGboost model presented a higher AUC for distinguishing ICM and DCM than all other models (LR, RAN and NNET). AUC: area under the curve; LR: logistic regression; NNET: neural network algorithm; RAN: random forest; XGboost: extreme gradient boosting

For the classification of ICM versus DCM, the performances of 4 ML models using all 16 features are presented in Tables 2 and 3; Figs. 4 and 5. For the XGBoost model, the average F-score was 0.73, with a sensitivity of 72%, specificity of 78%, the average accuracy of 75%, and AUC of the optimal subset of 0.934 (Table 2; Fig. 4). This model also showed good differentiation of ischemic/dilated cardiomyopathy in the externally validated cohort (AUC = 0.804, Fig. 5), with a sensitivity of 64% and a specificity of 93% (Table 3). Among the other models, the logistic regression model (AUC: internal verification 0.925, external verification 0.750), artificial neural network model (AUC: internal verification 0.893, external verification 0.780), and random forest model also had good discriminant performance (AUC: internal verification: 0.900, external validation: 0.761) (Fig. 5).

**Table 2:** Prediction of ICM and DCM in the test sample

Model	Accuracy	Sensitivity	Specificity	Precision	F1
RAN	0.73 ± 0.07 (0.60–0.85)	0.71 ± 0.14 (0.45–0.90)	0.76 ± 0.09 (0.60–0.90)	0.75 ± 0.07 (0.65–0.88)	0.72 ± 0.09 (0.53–0.84)
NNET	0.73 ± 0.02 (0.70–0.78)	0.67 ± 0.08 (0.58–0.84)	0.79 ± 0.08 (0.65–0.94)	0.75 ± 0.08 (0.67–0.93)	0.71 ± 0.05 (0.63–0.77)
LR	0.76 ± 0.08 (0.65–0.90)	0.76 ± 0.11 (0.60–0.95)	0.76 ± 0.10 (0.60–0.95)	0.77 ± 0.10 (0.65–0.95)	0.76 ± 0.08 (0.65–0.90)
XGBOOST	0.75 ± 0.06 (0.63–0.87)	072 ± 0.12 (0.60-1.00)	0.78 ± 0.07 (0.65–0.88)	0.76 ± 0.06 (0.71–0.90)	0.73 ± 0.08 (0.62–0.88)

Average of 10-fold cross-validation results shown by mean ± standard deviation

Abbreviations: LR: logistic regression; NNET: neural network algorithm; RAN: random forest; XGboost: extreme gradient boosting

**Table 3:** Prediction of ICM and DCM in the external validation

Model	Accuracy	Sensitivity	Specificity	Precision	F1
RAN	0.75	0.59	0.90	0.85	0.70
NNET	0.76	0.61	0.9	0.86	0.72
LR	0.74	0.59	0.9	0.85	0.7
XGBOOST	0.78	0.64	0.93	0.89	0.75

Abbreviations: LR: logistic regression; NNET: neural network algorithm; RAN: random forest; XGboost: extreme gradient boosting

### Calibration of the prediction models

Calibration was performed on this two-class classification task (identification of ICM versus DCM) for evaluating class assignment probability distribution. The XGBoost model’s Brier score, which measures the accuracy of the probabilistic predictions, for predicting cardiomyopathy in the optimal training set was 0.177, and that for the test set was 0.164, indicating that the ML-based model was well fitted and had good stability. Table 4 summarizes the Brier scores for the additional models.

**Table 4:** Summary of Brier scores for evaluating the calibration of the ML models (NNET, XGBoost, and RAN) as well as that of LR.

Model	Brier Score Training set	Test set
RAN	0.027	0.144
LR	0.158	0.116
NNET	0.141	0.169
XGBOOST	0.177	0.164

Abbreviations: LR: logistic regression; NNET: neural network algorithm; RAN: random forest; XGboost: extreme gradient boosting

## Discussion

Here we present, to the best of our knowledge, the first accurate and robust diagnostic classification method for the differential diagnosis between DCM and ICM, which we developed using four different supervised ML algorithms. Compared with the traditional diagnostic methods, the use of ICM and DCM clinical models provides a powerful ML framework for distinguishing similar clinical manifestations between these two types of cardiomyopathy.

### Previous literature

The high prevalence of HF and its association with diminished quality of life support the need for targeted treatment. The existing methods require certain techniques and equipment, which limits their clinical application for rapid diagnosis and effective treatment. In addition, it has been shown that in patients with new onset HF, the detection deficiencies identified in the study analysis were not limited to ischemic detection, indicating a larger problem that patients hospitalized with new HF may not have received appropriate HF testing [26]. In an increasingly cost-conscious era, there are concerns about overtesting in low-risk patients, and there is clearly underutilization of appropriate tests in high-risk patients. The use of ML algorithms for big data analysis in clinical research contributes to the development of widely applicable predictive models. As a non-invasive, low-cost and highly accurate tool, ML models may also help to better balance the relationship between risk assessment, cost-effectiveness and related testing, further helping community hospitals transfer patients most at risk or with a high probability of ICM to second-tier facilities, improving the detection rate of HF etiology, and strengthening timely referral management [27]. Previous studies have generated various models, including those to predict the readmission rate or death due to HF as well as prognosis of HF, and a diagnostic model for early recognition of HF. However, there are still some gaps in establishing a diagnostic model for predicting the etiology of HF [28–31]. ICM and DCM are both common causes of HF [4]. In a previous study for the classification and prediction of cardiomyopathy, Alimadadi et al. developed a ML prediction algorithm based on cardiac transcriptomic data [32]. The enrolled patients include 41 DCM patients, 47 ICM patients and 49 non-HF controls. The selected variables included related genes with strong contributions to cardiomyopathy. The model accuracy for differentiating ICM from DCM was as high as 85%. However, it may be difficult to apply in the clinic because of the difficulty in obtaining variables. In another study including 25 ICM patients, 13 DCM patients, and 30 elderly controls, Rodriguez et al. developed a ML model based on the analysis of electrocardiography (ECG) and blood pressure signal data [33]. The selected variables included the time series of beat-to-beat intervals (from the ECG signal) and end-systolic and diastolic pressure amplitude (from the BP signal). The best accuracy of the model for differentiating ICM from DCM was 84.2% [33]. However, the small sample size included in this study reduces the significance of the findings.

### Discussion of main results

Studies have shown the potential for the improved diagnostic performance of ML algorithms for cardiomyopathy; however, their application has been flawed due to the difficulty in obtaining clinical results and the characteristics of patients from various examination methods. In the present study, we developed a recognition ML algorithm by using only easy-to-obtain, highly sensitive and specific echocardiographic features and compared the diagnostic performances of four ML models on an unknown dataset. We found that four ML algorithms, including NNET, RAN, the integrated enhancement algorithm XGboost, and LR, all performed well in predicting and classifying ICM and DCM, which may be attributed to the high diagnostic value of the original data itself. Among these four models, the XGboost algorithm had the highest discriminant performance. The results from both internal verification and external verification in our study support the notion that ML can integrate a number of variables to build advanced algorithms that distinguish ICM from DCM. In addition, we also found clinically important factors related to the two kinds of cardiomyopathy in a ML model, including wall motion abnormalities. From physiological and pathological studies, in DCM patients with myocardial extensive diffuse damage, with tension reducing myocardial relaxation the four cardiac chambers were expanded, showing diffuse ventricular wall motion abnormalities. Coronary vessel distribution in ICM patients is associated with myocardial ischemia, and heart damage is limited to one side of the left ventricle, generating segmental disturbance of ventricular wall movement. The clinical consensus is that the difference in ventricular wall motion between the two groups has differential diagnostic significance, which is basically consistent with the structure of our model and also proves the reliability of ML. Although the data integration process is complex, clinicians can still readily understand the final outcomes of the analysis obtained from using the prediction model.

The four different ML algorithms used different structures to predict the end point. Each model also ended up correctly predicting different patients, and thus, the important variables in the XGBoost model were different from those in the RAN, LR and NNET models, as seen in Fig. 3. Hence, different algorithms can show efficient mutual complementation. As a result, many researchers have attempted to combine prediction algorithms to achieve a high degree of predictive accuracy [34, 35]. This approach, which is referred to as ensemble algorithms, is the next frontier in our studies.

### Clinical implications

Compared with traditional diagnostic methods, ML models serve as platforms for integrating multiple types of information [35], which may be more useful than one standardized interpretation of the data [36]. ML approaches do not underestimate the contribution of traditional echocardiography in the identification of cardiovascular diseases; rather, it provides a modern solution to integrate increasing numbers of parameters into the clinical database, thus simplifying the diagnostic process [37]. Considering the complexity of cardiovascular phenotypes and associated comorbidities, the potential of ML algorithms in personalized diagnosis and treatment cannot be overlooked [18]. Previously, we established a data-driven diagnostic system to accurately classify individual cases [38]. Despite the complexity of the data process, this process can be automated and easily performed in primary hospitals with limited conditions.

### Limitations

Despite the outstanding strengths of the novel prediction model generated in this study, there are some limitations in our study. First, although we fit the ML algorithm by determining the weight of each variable, we were not able to explain the algorithm in terms of clinical end point decision making. For instance, if a patient was predicted to have ICM by the ML algorithm in this study, the reason for the prediction could not be determined. Hence, explaining ML remains to be explored. In addition, this study did not include a comparative analysis of expert inspection results and machine results, which is a very important limitation. However, the model developed in our study can provide a valuable reference for primary-level hospitals with limited resources and conditions, as well as for inexperienced novice doctors and outpatient physicians who are not cardiac specialists. Current practice relies on the clinician’s interpretation of echocardiograms of heart failure patients, which is a subjective judgment based on the clinician’s knowledge and experience. Therefore, the findings cannot be quantified and integrated into any quantitative estimate for risk stratification. In contrast, machine learning models facilitate automated interpretation and risk quantification of echocardiograms, reduce variability and cost between human observers, and minimize variation in access to medical service. We see the primary role of the developed model as complementing rather than replacing the existing work of clinical teams. However, a prospective randomized study is indeed needed in the future to evaluate and compare the clinical outcomes achieved with the use of ML- and human-guided diagnostic strategies to adapt clinical practice applications. Second, because the analysis included patients of single race and was conducted in only two hospitals in China, this study may be subject to selection bias. It is necessary to validate this model in patients with ischemic/dilated cardiomyopathy in other countries. In addition, although the developed model was validated in test sets and external validation queues, the possibility of overfitting cannot be completely ruled out. Third, patients were assigned to the ischemic CM group based on the burden of coronary artery disease as assessed by invasive coronary angiography and prior medical history. However, a recent cardiac MRI study found that 11.8% of patients were traditionally classified as having ischemic CM (via angiography) and 1.5% of patients with non-ischemic CM showed a mixed CM pattern on MRI [39]. Therefore, there may also be a subset of patients in our cohort with mixed CM, which is inconsistent with the binary results of our model. A mixed CM pattern may lead to false positives or false negatives in our model, thus reducing its prediction accuracy. Fourth, the sample size of this study was small and included patients with WMAs. WMAs account for a large proportion in both clinical consensus and model structure, making a significant contribution to the classification of both. However, this group of patients is more difficult to classify. Future studies may require a larger sample size and exclusion of this group of patients to provide more reliable results for clinical practice. Fifth, differences between the two may have an impact on judgment, including clinical diagnosis and machine diagnosis, such as different ranges for LVEF, ventricular volume, and inner diameter. We excluded patients with 40–45% LVEF to reduce such effects, but still could not completely avoid them. Sixth, we can try to explore other features, including more advanced and accurate non-invasive imaging data, which can further improve the prediction accuracy of the model, such as exercise echocardiography, PET, CT, and CMRI features [40]. Exercise echocardiography can differentiate VDIC from DM with a reasonable degree of diagnostic precision, measuring changes in overall and regional systolic function with exercise, but the relevant research is largely dependent on the technique used, which in turn requires an observer with extensive experience [41]. Although CT can be used to evaluate perfusion [42], left ventricular function [43], and scarring [44], it is mainly used for coronary artery imaging in the clinic. Especially in patients with chronic CAD, CT may be restricted by a high calcium load. Dedicated cardiac PET-CT systems can simultaneously assess the presence and extent of coronary artery anatomy, ischemia, and hibernation myocardium. Similarly, this approach may be hampered by high cumulative effective doses of ionizing radiation [45]. Studies have shown that CMR is useful for ventricular volume and function evaluation as well as scar visualization, with a high degree of accuracy in assessing systolic reserve and ischemia in a single examination in the case of no exposure to ionizing radiation [46]. The ML framework discussed herein relies on data-driven echocardiographic diagnosis, which opens up a promising frontier for a cardiomyopathy classification system. In the near future, we will attempt to include more comprehensive information, and multi-dimensional and high space data are expected to produce better results and increase the generalization of groups. Seventh, instead of using the original echocardiographic image signals, we used the features. Our goal was to make the ML model easier to use in general clinics and to achieve high-precision predictions under limited conditions. However, this approach may potentially limit the performance of the recognition algorithm. In our future studies, we plan to use raw echocardiographic image signals to predict cardiomyopathy.

## Conclusion

We generated ML prediction models based on the parameters derived from echocardiography to differentiate ICM- and DCM-induced HF. Our study provides evidence that ML algorithms using demographic and echocardiographic features can distinguish ICM and DCM and have the potential for application in precision medicine in the clinic, providing a new possibility and reference for the etiological diagnosis of HF.

## Data Availability

The datasets generated and analyzed during the current study are not publicly available due to none of the data types requiring uploading to a public repository but are available from the corresponding author on reasonable request.

## Abbreviations

ICM: ischemic cardiomyopathy
DCM: dilated cardiomyopathy
ML: machine learning
CM: cardiomyopathy
HF: heart failure
CAD: coronary artery disease
BMI: body Mass Index
LAD: left atrial diameter
LVEDD: left ventricle end-diastolic diameter
LVESD: left ventricle end-systolic diameter
LVPWT: left ventricle posterior wall thickness
DWMA: diffuse ventricular wall motion abnormalities
SWMA: segmental ventricular wall motion abnormalities
LVEF: left ventricle ejection fraction
E/A: the ratio of peak E to peak A
FS: left ventricle fractional shortening
SV: stroke volume
MR(m-s): moderate to severe mitral regurgitation
MVR(m-s): moderate to severe multiple valve regurgitation
CO: cardiac output
LR: logistic regression
NNET: neural network algorithm
RAN: random forest
XGboost: extreme gradient boosting
AUC: area under the curve
ECG: electrocardiography
